# Trichotemnomania in an Adolescent Girl: A Case Report of an Asian Child and Literature Review

**DOI:** 10.1155/2020/6615250

**Published:** 2020-12-08

**Authors:** V. Thadchanamoorthy, Markandu Thirukumar, Kavinda Dayasiri, N. Thamilvannan, Judy Jeyakumar

**Affiliations:** ^1^Faculty of Health Care Sciences, Eastern University, Sri Lanka; ^2^Base Hospital, Mahaoya, Sri Lanka; ^3^Teaching Hospital, Batticaloa, Sri Lanka; ^4^Department of Psychiatry, Base Hospital, Valaichenai, Sri Lanka

## Abstract

Trichotemnomania (TT) refers to cutting or shaving of one's own hair as a compulsive act. This condition is reported rarely and may be indicative of an underlying obsessive-compulsive disorder. TT may be misdiagnosed with trichotillomania or other disorders such as alopecia areata, tinea capitis, and postinflammatory scars. The diagnosis of trichotemnomania is confirmed by dermoscopic assessment, histopathological changes of hair, and correlation of these findings with clinical history. A fourteen-year-old adolescent girl presented with focal hair loss over forehead for duration of two-weeks and periodic abnormal breathing and poor sleep for 2-month duration. Besides, she had also lost some of pubic hair and hair on the forearm over preceding 24 hours. This patient was assessed by a team including a paediatrician, gynecologist, dermatologist, and psychiatrist to gather focused medical history and to perform physical examination, laboratory investigations, and dermoscopic assessment. It was revealed that she used to shave or cut regularly following stressful situations across various aspects of her life and hyperventilate as a means of relieving her stress. Eventually, she was diagnosed to have trichotemnomania and was started oral sertraline 50 mg/day for one month. Clinical features and her behaviour improved with regular cognitive behavioural therapy, and hairs were demonstrated to grow up normally with change in behaviour. Currently, she does well at school and is off medications and being followed up at the child guidance clinic. Trichotemnomania is a very rare disorder which is characterised by cutting or shaving of one's own hairs as a compulsive habit. The condition needs careful and detailed assessment by a team of specialists to identify coexisting psychiatric disorders and offer treatment.

## 1. Background

Trichotemnomania (TT) is an extremely rare and underassessed condition [1]. It is defined as shaving or cutting of one's own hair anywhere in the body using scissors or a razor, and it has been considered as obsessive-compulsive behaviour [2]. Although trichotillomania has been well-described since 1987 under the Diagnostic and Statistical Manual of Mental Disorders [1], trichotemnomania is rarely reported and likely misdiagnosed. The name trichotemnomania stems from Greek words: thrix (hair), temnein (cut), and mania (madness) [3]. In contrast to trichotemnomania, children with trichotillomania pull their own hairs. Both conditions are self-induced and not convinced to change their routine behaviour [4]. Children with this condition are ashamed due to their behaviour and have psychological pain in them [4]. Unless clinicians have a greater degree of suspicion and perform dermoscopic examination, there is a high probability that the diagnosis remain unexposed and may be misdiagnosed as trichotillomania. The dermatoscopic examination demonstrates healthy-looking hair shafts and is helpful to differentiate trichotemnomania from other similar conditions mimicking the morphological appearance yet with a different pathological diagnosis [4–6]. Only a very limited number of cases with trichotemnomania have been reported in medical literature including three adolescent children [2–7]. The authors present an adolescent girl who had the habit of cutting and shaving her own hair as a means of relieving stress, and the diagnosis of trichotemnomania was confirmed based on clinical history, dermoscopy examination of hair, and histopathological examination by a multidisciplinary group of specialist health professionals. The reported child also was diagnosed to have obsessive-compulsive disorder and currently being followed up in the child guidance clinic under the same multidisciplinary team.

## 2. Case Presentation

A 14-year-old previously healthy child was referred to the paediatrician by the gynecologist as she had unexplained hair loss on the left side of forehead for two-week duration. She did not have hair loss at any other sites of her body initially. In addition, parents also reported that she had poor sleep at night and also had witnessed abnormal fast breathing from time to time for two months. Her school performance was above average. Family history revealed that her parents were elderly and overprotective but had no similar illness or any other psychiatric illnesses in the family. The patient reported that parents had never allowed this child to have friends or share her problems with friends. Except during school hours, she had no association with her friends. As there was the pandemic of COVID-19, she did not attend school and was missing her school friends.

**Figure 1:**
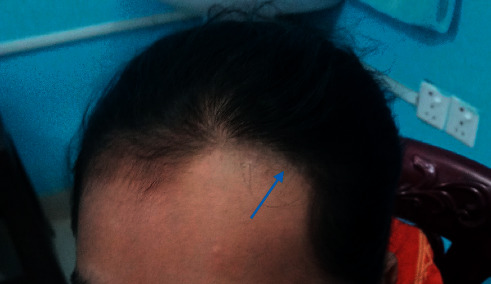
Hair loss over the forehead.

**Figure 2:**
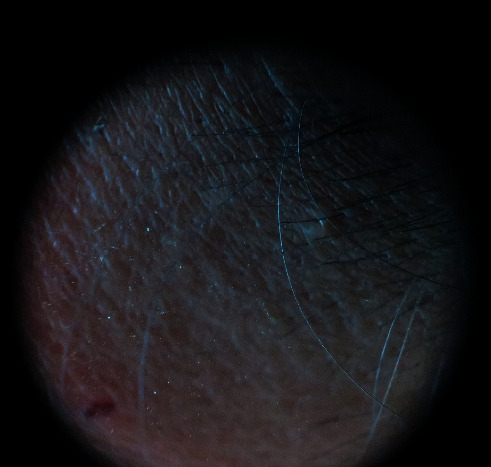
Dermoscopic appearance of forehead.

**Figure 3:**
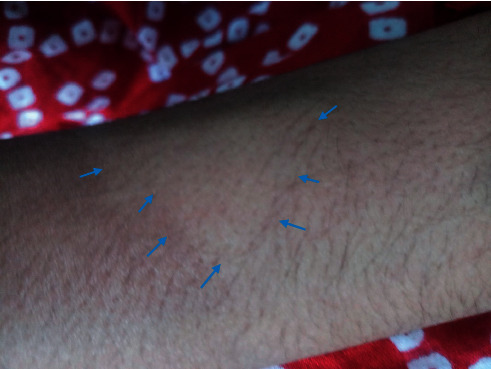
Dermoscopic appearance of forearm.

Physical examination revealed that she is active, pink, and well-grown. There was oval-shaped patch of hair loss on the left forehead (Figure 1). No other hair loss was noted. All other systems examination was normal. The dermatologist suspected the possibility that hair had likely been shaved rather than pulled (trichotillomania) or lost spontaneously. The dermoscopy examination revealed uniform loss of hair with all the hair follicular openings filled with black hair shafts (Figure 2). Furthermore, there was no erythema, tenderness, ulcers, or scaring, and it was confirmed that hair loss was caused by shaving with a blade upon careful history taking. The hair-pull test was negative. Her parents were advised about the nature of her problem, but parents were not convinced at all initially. She was referred to the psychiatrist for further assessment. While waiting for psychological assessment in one-week time, her mother again reported that there was hair loss in the pubic region and hand on the next day (Figure 3). These patches of hair loss were also confirmed to be secondary to shaving rather than pulling of her hairs. The child denied the act of shaving. However, parents were convinced this time and were advised to observe the child as to whether she had any shaving razors or been using her father's shaving razor. Subsequently, the mother could find a razor in her bed room.

The psychiatrist further assessed her and found that she had clinical features of obsessive-compulsive disorder and anxiety behaviour. The final diagnosis was made as trichotemnomania secondary to obsessive-compulsive disorder following multidisciplinary assessment. She was started on sertraline at a dose of 50 mg/day and behaviour modification counselling.

After 3 months of treatment, the medication could be tailed off. Currently, she has been under follow-up at the child guidance clinic and off all medications. As school had been restarted following control of COVID-19 outbreak in the country, she now has an opportunity to make new friends. Her hyperventilation and sleep disorder have also resolved.

## 3. Discussion

The reported patient had obsessive-compulsive disorder, which is defined as having recurrent, persistent, irresistible thoughts, ideas, impulses, or sensations (obsessions) that make one to feel doing an act repeatedly (compulsive behaviour) to relieve anxiety [8]. Trichotemnomania is induced by anxiety with purpose of avoiding stress. Trichotemnomania is defined as hair loss precipitated by disturbing thoughts and excessive emotional turmoil as patient tries to stop the action and feeling of satisfaction when they do the action [9]. Similar act has been noticed in trichotillomania [10]. These patients may feel ashamed due to their act and appearance, and also they may have feelings of doing this act as a mistake [11]. Patients with trichotemnomania usually have a fair degree of insight [12].

The reported child denied the act which made her parents to consult various specialist health professionals, and clinicians suspected various causes for her hair loss including endocrinological aetiologies. Unexplained symptoms have the potential to make clinicians to perform unnecessary investigations including invasive procedures such as biopsy to exclude other possible differential diagnosis [4–6]. All the possible differentials including systemic lupus erythematous and endocrine abnormalities were ruled out in this child.

As reported in literature [7], this child did not worry about her hair loss and suggested interventions unlike her parents who were worried about their only daughter's future including marriage and physical appearance. She kept on cutting pubic hair and forearm hair even after her first consultation with paediatrician. She reported to her mother that she had hair loss in other sites the following day but denied that she shaved hair. Her act of rejection of the fact that she shaved might be due to feelings of guilty and discomfiture. Similar observations have been made in other reports [6]. Fortunately, she was not commenced on any medications although some clinicians suggested treatment with hematinics at the initial stage of her presentation.

The literature reveals that patches of hair loss in trichotemnomania are often seen in scalp, although it is possible that other sites can be involved including pubis, eye lashes, eyebrows, and axilla [6, 13]. When hair is cut or shaved, dermatoscopy shows presence of the follicle opening with filled hair shaft and an otherwise healthy-looking scalp with no signs of inflammations such as erythema, tenderness ulcers, and scaring. Hair-pull test is negative for trichotemnomania [3, 4]. Hairs preserve normal pigmentation and texture on the cleanly cut surfaces [14]. The reported child had lesions in forehead, followed by pubic area and forearm, and the dermatoscopic findings are compatible with trichotemnomania.

The differential diagnosis of trichotemnomania includes trichotillomania, trichoteiromania, trichodagonomania, and alopecia areata which all share similar phenotypic appearance, although histopathological findings are different. The only way to differentiate was either dermatoscopic assessment or histopathological assessment [2].

Trichotillomania is common in scalp and occurs also in other sites [6, 13]. The physical appearance comprises irregular and bald patches containing varying lengths of hairs, and histology reveals an increased number of catagen hair follicles with pigment cast, follicular plugging, and trichomalasia [4, 15]. Trichoteiromania is described as hair loss secondary to psychiatric disorders. The affected patient tries to break their hairs by rubbing or scratching of hairs [16]. Their hairs appear as bald spots with different hair lengths as if they were cut by scissors, and white tips associated with distal splitting is a histological feature. The appearance is described as brush-like spitting at the end of the hair shaft on the light microscopy [16, 17]. Tricodagonamania is also another psychiatric disorder, which leads to compulsive habit of biting their own hair and hair loss is not located in the head, and therefore, no loss of hair is present in scalp [18]. Alopecia areata is another differential diagnosis and occurs secondary to autoimmune disease. It can affect any part of the body with patchy boldness over a period of time. Hair-pull test would be positive in alopecia areata. Trichoscopy examination shows yellow dots (hyperkeratotic plugs), small exclamation-mark hairs, and black dots (destroyed hairs in the follicle opening) [19]. Hairs upon regrowth appear to be blonde or white hairs with no pigmentation [14]. Tinea capitis present with single or multiple patchy hair loss secondary to cutaneous fungal infection. It usually accompanies inflammation, scaring, pustules, and itching often leaving black dots. Wood-lamp examination and microscopy can identify the organism [20, 21]. The postinflammatory and posttraumatic scars can also mimic the trichotemnomania following patchy infection or trauma.

The management of obsessive-convulsive disorder prevents the act of compulsive cutting of hairs. The main treatment modality is cognitive-behavioural therapy combined with pharmacological treatment to control obsessive-compulsive behaviour [22]. The reported child was started on both drug treatment and cognitive behavioral therapy. She responded to treatment successfully over 3 months.

Trichotemnomania is a rare, yet underdiagnosed and underreported, condition according to available literature. The clinicians should explore all children presenting with unexplained focal loss of hair in-depth by focused history, and physical examination before an extensive list of investigations would be carried out to arrive at a diagnosis of an underlying organic disorder. The management of trichotemnomania in children is multidisciplinary and involves paediatrician, dermatologist, psychiatrist, and psychologist.

## Data Availability

The data that support the findings of this case report are available from Medical Records Department, Batticaloa Teaching Hospital, but restrictions apply to the availability of these data, which were used under license for the current report and so are not publicly available. Data are, however, available from the authors upon reasonable request and with permission of Medical Records Department, Batticaloa Teaching Hospital, Sri Lanka.

## Abbreviations

COVID-19: Coronavirus disease-2019
FSH: Follicular stimulating hormone
LH: Luteinizing hormone
ANA: Antinuclear nuclear antibody.
